# Stimulation of Bone Healing by Sustained Bone Morphogenetic Protein 2 (BMP-2) Delivery

**DOI:** 10.3390/ijms15058539

**Published:** 2014-05-14

**Authors:** Mirja Faßbender, Susann Minkwitz, Catrin Strobel, Gerhard Schmidmaier, Britt Wildemann

**Affiliations:** 1 Julius Wolff Institute, Center for Musculoskeletal Surgery, Charité-Universitätsmedizin Berlin, Augustenburger Platz 1, Berlin 13353, Germany; E-Mails: mirjafassbender@web.de (M.F.); susann.minkwitz@charite.de (S.M.); post@catrinstrobel.de (C.S.); 2 Berlin-Brandenburg Center for Regenerative Therapies, Charité-Universitätsmedizin Berlin, Berlin 13353, Germany; 3 Berlin-Brandenburg School for Regenerative Therapies, Charité-Universitätsmedizin Berlin, Berlin 13353, Germany; 4 Department of Orthopedics, Orthopedics and Traumatology, Heidelberg University Hospital, Schlierbacher Landstraße 200a, Heidelberg 69118, Germany; E-Mail: gerhard.schmidmaier@med.uni-heidelberg.de

**Keywords:** sustained bone morphogenetic protein-2 (BMP-2) release, implant coating, impaired bone healing, micro-computed tomography (μCT), histology, biomechanical testing

## Abstract

The aim of the study was to investigate the effect of a sustained release of bone morphogenetic protein2 (BMP-2) incorporated in a polymeric implant coating on bone healing. *In vitro* analysis revealed a sustained, but incomplete BMP-2 release until Day 42. For the *in vivo* study, the rat tibia osteotomy was stabilized either with control or BMP-2 coated wires, and the healing progress was followed by micro computed tomography (μCT), biomechanical testing and histology at Days 10, 28, 42 and 84. MicroCT showed an accelerated formation of mineralized callus, as well as remodeling and an increase of mineralized/total callus volume (*p* = 0.021) at Day 42 in the BMP-2 group compared to the control. Histology revealed an increased callus mineralization at Days 42 and 84 (*p* = 0.006) with reduced cartilage at Day 84 (*p* = 0.004) in the BMP-2 group. Biomechanical stiffness was significantly higher in the BMP-2 group (*p* = 0.045) at Day 42. In summary, bone healing was enhanced after sustained BMP-2 application compared to the control. Using the same drug delivery system, but a burst release of BMP-2, a previous published study showed a similar positive effect on bone healing. Distinct differences in the healing outcome might be explained due to the different BMP release kinetics and dosages. However, further studies are necessary to adapt the optimal release profiles to physiological mechanisms.

## 1. Introduction

Fracture healing is a complex physiological and a temporally coordinated process of cells, growth and differentiation factors, hormones, cytokines and extracellular matrix interactions. The healing process can be divided into three phases: inflammation, repair and remodeling [1]. The initial inflammatory phase is mainly characterized by non-specific wound healing pathways combating infection, removing cell debris and organizing the fracture hematoma. Subsequently, signaling pathways controlling tissue regeneration and remodeling are activated. Fibrous tissue and cartilage formation followed by primary bone formation and cartilage resorption are mainly guided by the expression of members of the transforming growth factor TGF-β superfamily, like bone morphogenetic proteins (BMPs) [2]. Although different BMPs are closely related in structure and function, they exhibit different temporal patterns of expression at different stages of fracture healing. In particular, BMP-2 plays a key role influencing chondrogenesis and osteogenesis [3,4], as well as re-vascularization [5]. BMP-2 is considered essential in fracture healing, since Tsuji *et al.* demonstrated that mice with impaired BMP-2 expression showed normal skeletal development, but impaired fracture healing, and although other BMPs could compensate for the lack of BMP-2 during bone development, none are able to substitute for the function of BMP-2 during bone healing [6]. Therapies for bone regeneration using cytokines with bone-inducing activities, such as BMPs, basic fibroblast growth factor, vascular endothelial growth factor, platelet-derived growth factor or insulin-like growth factor have recently attracted attention [7]. BMP-2 and BMP-7 have been approved for clinical use [8,9]. However, early diffusion, absorption of single dosages or a temporally inappropriate application may limit the bone inductive effects or may even demand higher dosages. A prolonged and controlled delivery of growth factors would offer the chance to adapt treatment strategies to physiological expression patterns of the specific factors and, therefore, BMP-2 treatment could be more efficient for the stimulation of healing. It has been proven in various animal models that the BMP-2 signaling cascade starts the early moments of the initial phase of bone healing, triggering the inflammatory response and periosteal activation. However, BMP-2 is also important during later phases of chondro- and osteogenesis [10–13]. Experimental models testing the effect of a time-delayed BMP-2 application either by using void filling materials [14,15] or adenoviral vector [16] showed promising results. In a previous study, Strobel *et al.* [17] demonstrated the possibility to achieve a sequential and delayed release of growth factors from a one-component polymeric implant coating. As a follow-up the present study investigated the effect of a sustained BMP-2 release from a poly(D,L-lactide) (PDLLA) implant coating on bone healing in an animal model showing impaired bone healing. The impaired healing model was established and described in a previous study [18].

## 2. Results and Discussion

### 2.1. Results

#### 2.1.1. *In Vitro* Release Kinetics

**Figure 1.**
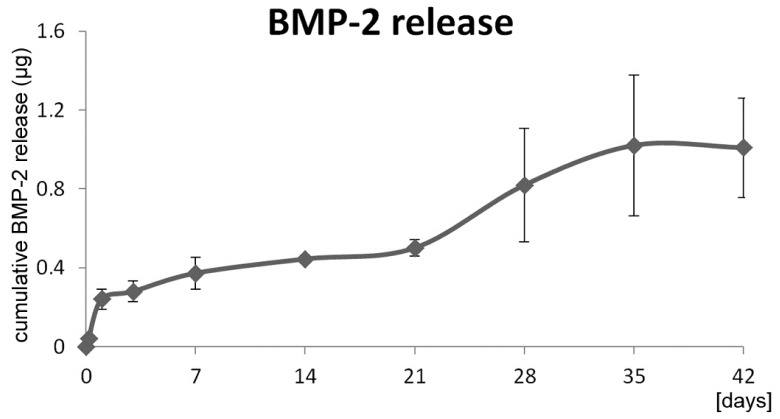
Cumulative bone morphogenetic protein 2 (BMP-2) release from the poly(D,L-lactide) (PDLLA) implant coating (*n* = 3). Mean values with standard deviation are depicted.

*In vitro* elution studies showed a sustained release of the incorporated BMP-2. The weak burst release within the first day was followed by a sustained release of approximately 1 μg BMP-2 in total until Day 42 (Figure 1).

#### 2.1.2. Micro-Computed Tomography Evaluation

**Figure 2.**
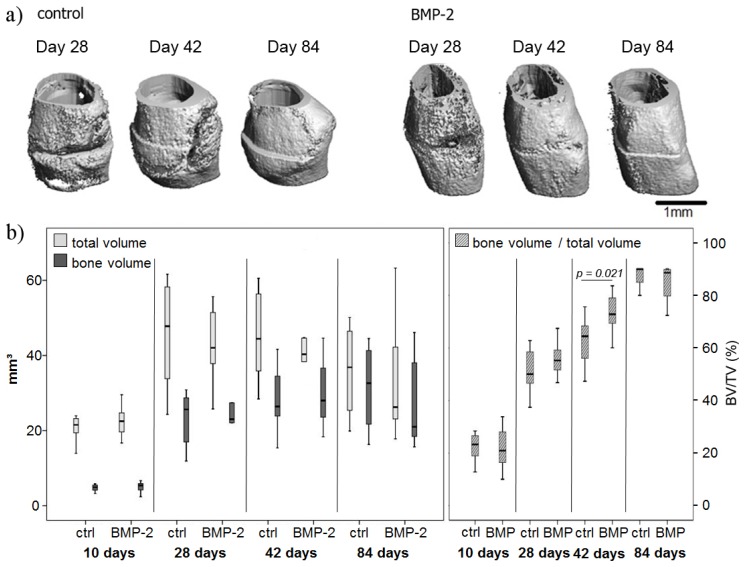
(**a**) μCT 3D reconstructions of selected tibiae of the control and the BMP-2 group over the healing time points. The scans were performed with the Viva40 μCT (Scanco) with a voxel size of 25 μm. The cortical bone has been removed. Scale bar: 1 mm; (**b**) Results of the μCT analysis of the control (ctrl) and the BMP-2 treated groups. The first graph shows the bone volume and total volume (total callus volume, including bone volume), and the percentage amount of mineralized bone in the total callus region is depicted in the second graph. The bone volume/total volume (in %) was significantly increased at Day 42 in the BMP-2 group compared to the control group.

The 3D reconstruction of specimens exemplary chosen showed an increasing callus mineralization over time in both control and BMP-2 treated groups (Figure 2a). At Day 84 the callus volume decreased in both groups, but slightly more in the BMP-2 treated animals.

The μCT data revealed an increase in callus size from Day 10 to 28 and a decrease from Day 28 to 84 in both groups (Figure 2b). Between Days 28 and 84, the total callus volume tended towards a reduction in the BMP-2 group, but not to a significant extent. At Day 10, approximately a fifth of the total tissue was mineralized in both groups without a significant difference. Over time, the amount of mineralized tissue increased, resulting in nearly 90% mineralized tissue in the total callus (Figure 2b). The mineralization of the callus was significantly higher in the BMP-2 group compared to the control at Day 42 (*p* = 0.021).

#### 2.1.3. Histology and Histomorphometry of the Healing

At Day 10, the periosteal callus tissue of both groups consisted of inflammatory cells, reparative granular cells (fibroblasts), chondrocytes and early woven bone. The callus area and amount of cartilage was comparable between both groups.

**Figure 3.**
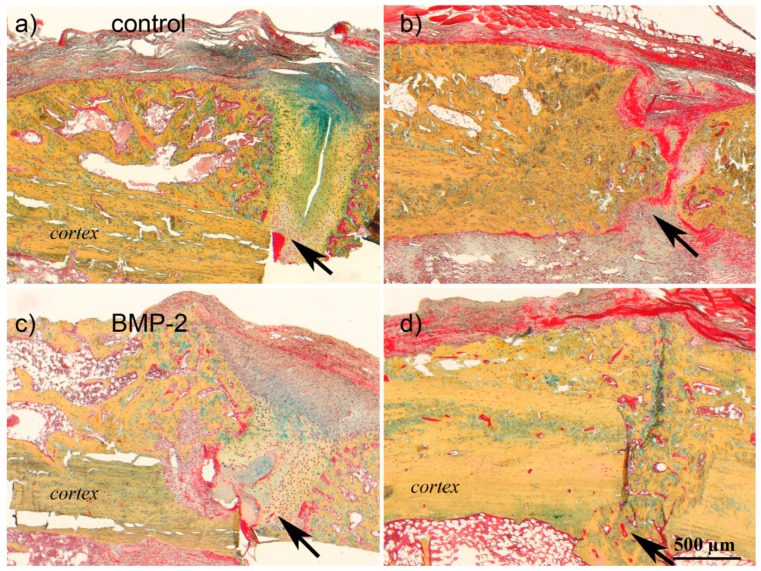
Histological staining of the calluses of the control and the BMP-2 group at Days 28 and 84. Movat pentachrome stainings of the two groups (control group: **a**,**b**; and BMP-2 group: **c**,**d**) at Days 28 (**a**,**c**) and 84 (**b**,**d**). The arrows point to the osteotomy gap. At Day 28, the calluses of both groups showed no fully mineralized bridging, as fibrous tissue was still filling the gap above the osteotomy. After 84 days, the healing progressed with fully mineralized callus in the BMP-2 group. Scale bar: 500 μm.

Histological analysis (Movat pentachrome staining) revealed accelerated callus maturation in the BMP-2 group (Figure 3), which supported previous μCT data. At Day 28 in both groups, a prominent callus was visible consisting of mineralized woven bone adjacent to the cortex and cartilage and connective tissue within the osteotomy gap. At Day 42, the osteotomy gap was still filled with connective tissue in the control group, whereas in the BMP-2 group, the defect was bridged by mineralized woven bone. The amount of fibrous tissue in the gap was reduced at Day 84 in the control group, with an increase in mineralized tissue, but mineralized bridging was not completed. In the BMP-2 group, less fibrous tissue was visible, and the remodeling of the woven bone was already initiated.

**Figure 4.**
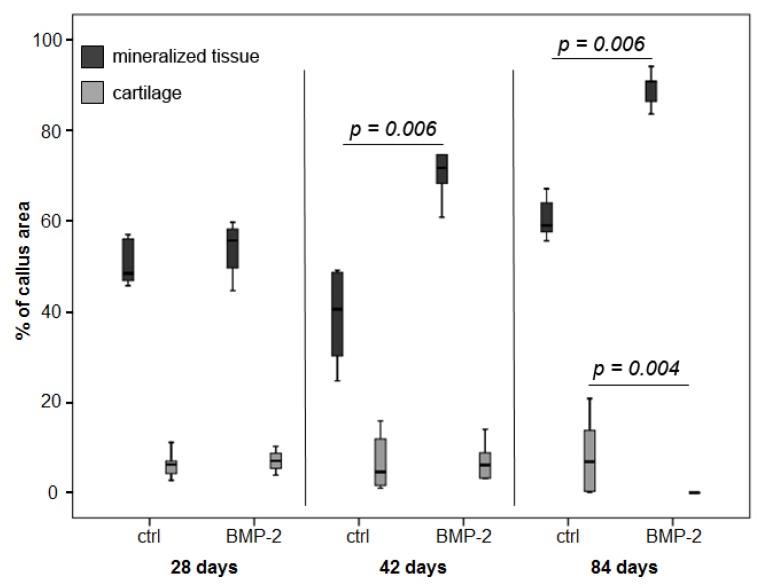
Histomorphometry-based data of the callus composition (mineralized tissue area and cartilage tissue area relative to the total callus area) over time. The mineralization of the callus was significantly enhanced in the BMP-2 group at Days 42 and 84 (*p* = 0.006), whereas the cartilage area was significantly reduced after BMP-2 treatment at Day 84 (*p* = 0.004).

The histomorphometrical analysis revealed a slight decrease in the total callus area from Day 28 to 84 in both groups. The amount of mineralized tissue in the callus decreased in the control group from 48.4% at Day 28 to 40.5% at Day 42, but then increased to 59.0% at Day 84. In the BMP-2 group, the amount of mineralized callus tissue increased constantly from 55.6% up to 90.8% at Day 84. A significant difference between the groups was found at Days 42 and 84. The cartilage proportion of the callus slightly decreased from Day 28 to 42 (6.2% to 4.6%) and afterwards slightly increased up to 6.9% in the control. In the BMP-2 treated animals, there was a constant decrease, and at Day 84, only one animal still had a small island of cartilage; in all other animals, cartilage was replaced by mineralized tissue (Figure 4).

#### 2.1.4. Biomechanical Testing

The osteotomized tibiae reached at no time point the mechanical properties of the intact contralateral tibiae.

The stiffness of the tibiae from the control group increased only slightly from Day 28 to 84 after osteotomy, not reaching more than 45% of stiffness of the intact contra-lateral tibiae.

**Figure 5.**
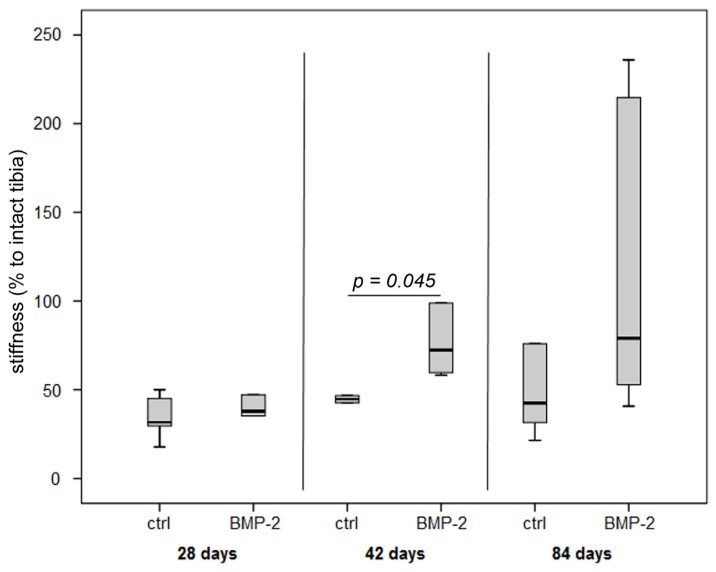
Results of the biomechanical testing of osteotomized tibiae expressed as normalized stiffness to the respective intact tibia.

In the BMP-2 group, values steadily rose and biomechanical strength reached 79% compared to the intact bone at Day 84, however, with a high variation. Values between both groups were significantly different at Day 42 (*p* = 0.045) (Figure 5).

### 2.2. Discussion

The present study investigated the effect of local and sustained release of BMP-2 on impaired osteotomy healing in a rat model. For drug delivery, a well-established polymeric implant coating was used and modified by varying the ratio of polymer/solvent/drug to realize a sustained drug release over at least 42 days. Healing was followed over a period of 84 days, and a significant improvement was seen, as shown by μCT, histomorphometry and mechanical testing.

A previous study used the same animal model and drug delivery system, but releasing the BMP-2 with an initial burst of approximately 50% within the first two days [19]. The evaluation of the healing showed a stimulation of the healing process, as seen by a significant higher stiffness and load after Days 28 and 42 accompanied by a higher mineralization at Day 42.

Comparing the sustained release of BMP-2 described in our study, with this previous study, the sustained release resulted in a slightly later improvement of the healing with a significant increase in the ratio of bone volume/total volume and the stiffness at Day 42 and a higher mineralization at Days 42 and 84. This slightly delayed stimulation of healing might be explained by the different release kinetic profiles. The initial burst release resulted in an early stimulation of the healing, whereas with the sustained release, a healing stimulation was more profound at the later time points. The different release kinetics were obtained by modifying the coating. The coating showing the burst release was made of 100 mg of PDLLA in 1.5 mL ethyl acetate and 5% of BMP-2 (*w*/*w* in poly(D,L-lactide)) resulting in 50 μg of BMP-2 per implant), whereas in the presented study, the amount of PDLLA was doubled and only half of the BMP-2 concentration was used (2.5% *w*/*w* in PDLLA, resulting in 40 μg of BMP-2 per implant). As shown in a previous study, the increase of total PDLLA leads to a thickening of the coating layer and, therefore, to a prolonged release, whereas the unreleased BMP-2 is still incorporated in the coating, as detected by the enzyme linked immunosorbent assay method [17]. This coating modification ensured different release kinetics with a similar drug load. However, the released dosage has to be considered. Using the burst release approach an approximate release of 80% BMP-2 is expected after 42 days [20], resulting in approximately 40 μg in that study. The sustained release, however, was not completed after 42 days, and only around 1 μg was released. The release experiment was performed with phosphate buffered saline as elution medium. A previous study showed that the use of cell culture medium resulted in an increased amount of released factors [17], and a different release *in vivo* might be expected, but is not proven. However, based on the obtained different released profiles, the much lower BMP-2 dosage used in this study showed a similar effectiveness than the higher burst release dosage used in the previous study. Even if this is a very extreme difference, the fact that a dosage reduction can be similarly effective at a higher dosage has been shown earlier. A study published as early as 1994 showed that the same healing result can be obtained with different dosages of osteogenic protein-1 (BMP-7) ranging from 6.15 to 400 μg used to fill a 1.5-cm segmental defect of the rabbit ulnar [21]. These extreme differences in the dosage are somehow comparable to the dosages used in the present and the previous study for the stimulated bone healing in that rat osteotomy model [1]. A current study also using BMP-7, but loaded onto a polycaprolactone scaffold for a 3-cm critical size tibia defect in a sheep model, showed that the lower dosage (1.75 mg) was as effective as the higher dosage (3.5 mg) [22].

Several experimental studies investigated the effect of the timing of growth factor delivery. The addition of BMP-2 at different time points after initial implantation of hydroxyapatite matrices revealed a less effective ectopic ossification compared to the simultaneous application of BMP and the matrices [23]. The implantation of the matrix four weeks before BMP application resulted in the weakest ossification and indicated that tissue already formed around the implant might have reduced the ability of applied BMP-2 to recruit mesenchymal progenitor cells from the surrounding to stimulate bone formation. A less delayed application (one week) revealed no significant difference compared to simultaneous application. Betz *et al.* [16] also used different application time points to investigate the effect of BMP on bone healing. They observed a higher incidence of bone union with greater bone mineral content and improved mechanical strength in animals receiving an adenoviral BMP-2 vector injection at Days 5 and 10 rather than intraoperatively or 24 h after the creation of a femoral critical size defect. The modification of drug release and dosage by different viral transfection methods (short term, high dosage: adenoviral; prolonged, low dosage: lentiviral) resulted in a trend towards the better healing of a femoral defect when the BMP-2 was expressed more prolongedly, but with a lower dosage [24]. Further Asamura *et al.* [14] used a dog model of orbital defects. Bone defects were filled either with a complex of BMP-2 saturated gelatin hydrogel encased by a substance-free biodegradable copolymer for a sustained release or with the copolymer directly saturated with the same amount of BMP-2 for an accelerated release. Those authors described an enhanced formation of new bone and improvement in defect healing after the usage of the slow release construct. A direct comparison of drug release kinetics on bone healing was carried out using BMP-2 absorbed to deproteinized bone (fast release) or by deproteinized bone bearing a coating-incorporated depot of BMP-2 (slow release) [25]. The slower release was more efficient than the faster release, shown by the histomorphometric analysis of the bone healing process.

The optimal time point for BMP stimulation, however, needs to be analyzed. A very detailed analysis of the expression of several members of the TGF-β superfamily revealed a very early expression of BMP-2 (Day 1 after fracture) that was followed by a continuously elevated expression level peaking again at Day 21 [10]. If a stimulation at two time points might be more effective compared to a more continuous delivery must be clarified in future studies.

Loading calcium phosphate cement with different concentrations of BMP-2 only, the higher concentration was sufficient to stimulate bone formation [26]. For the high concentration, polymeric microparticles loaded with 10 μg BMP-2 and, for the low concentration, loaded with 2 μg BMP-2 were used, mixed with cement and implanted in an 8-mm cranial defect. This study utilized *in vivo* imaging and found that only 30% of the incorporated BMP-2 was released after five weeks. This incomplete release was also expected in the present study based on the *in vitro* release experiments. The polymeric implant coating used in the present study has been investigated in detail over the last decade. The properties fulfil the requirements, such as mechanical stability, storability, good biocompatibility and the possibility, to incorporate various substances [20,27–30]. However, differences in the degradation processes of the coating between *in vitro* and *in vivo* studies are expected, and an *in vivo* study on the drug release may prove helpful in clarifying this issue.

Even if very different drug delivery approaches and alternative animal models were used, a prolonged application of a lower BMP amount seems to be as effective as higher BMP burst amounts. The release kinetics can be modified by various methods, as described above. Therefore, the right drug release system seems to be an important tool for the optimization of BMP therapy. The studies mentioned previously utilized either scaffolds for defect filling or adenoviral BMP vector injections. The greatest benefits of scaffolds is in filling defects, but if no space needs to be filled, a local and controlled release from an implant coating is a suitable alternative.

## 3. Experimental Section

If not stated otherwise, all companies or laboratories were located in Germany.

### 3.1. Polymer Coating of Titanium Kirschner-Wire (K-Wires)

The polymer, poly(D,L-lactide) (PDLLA, Boehringer Ingelheim, Ingelheim), was used as the drug delivery system for the coating of titanium Kirschner-wires (k-wires, 1 mm, Synthes, Oberdorf, Switzerland). Two-point-five percent of BMP-2 (Osteogenetics GmbH, Würzburg, Germany) was added to a PDLLA solution (200 mg/1.5 mL ethyl acetate, Sigma Aldrich, Taufkirchen, Germany), and the wires were coated by dipping twice, up to a length of 45 mm.

This resulted in a total amount of approximately 40 μg BMP-2 in the coating of the entire wire. The coated wires were stored sterile packed at −20 °C with desiccant to avoid humidity until usage. All steps were prepared under a laminar air flow and sterile conditions.

### 3.2. In Vitro Release Kinetics

*In vitro* release kinetics were performed after Strobel *et al.* [17]. Briefly, the coated wires were placed in 15-mL Falcon tubes with 5-mL sterile phosphate buffered saline (PBS plus 1% BSA, Biochrom GmbH, Berlin, Germany) completely covering the coating (*n* = 3). Samples (0.5 mL) were taken and analyzed at different time points up to eight weeks. The sample volume was substituted with fresh PBS. The elutions were performed in an incubator at 37 °C, 5% CO_2_ and 95% humidity, and BMP-2 was quantified using the BMP-2 ELISA construction-kit (Antigenix-America, Huntington, NY, USA). The cumulative release kinetics between the sampling time points were calculated.

### 3.3. Surgical Model

All animal experiments were approved by the local authorities (G0006/10) and complied with international legal regulations. Five-month old female Sprague Dawley rats (Charles River Laboratories International, Inc., Sulzfeld, Germany), weighing 250–280 g, were used. The osteotomy model has been described in a previous paper [18]. Briefly, anesthesia was performed with isoflurane and by an intraperitoneal injection of a ketamine/xylazine mixture (80 and 12 mg/kg body weight, respectively). The right lower leg was shaved and disinfected. The medullary cavity of the tibia was opened and reamed twice. The tibia was osteotomized at the midshaft level using a diamond disk (HORICO, Berlin, Germany). For stabilization, a wire coated with substance-free PDLLA (control) or BMP-2 in PDLLA was inserted from the proximal end of the tibia into the medullar canal. The fibula was fractured manually. The wound was closed, and gentamycin ointment was applied locally. For pain prophylaxis, the animals received buprenorphine (0.05 mg/kg body weight subcutaneously) for the first 3 days after the intervention. Euthanasia was performed in deep anesthesia by an intracardiac injection of potassium chloride.

### 3.4. Radiography and Micro Computed Tomography (μCT)

After anesthesia with isoflurane and intraperitoneal injection of a ketamine/medetomodin mixture (10 and 0.15 mg per animal, respectively), the rats were placed in a custom-made scanning bed for the μCT analysis. The right leg was fixed by adhesive tape strips to ensure horizontal positioning of the tibia. A Viva 40 μCT (Scanco medical AG, Brüttisellen, Switzerland) was used to scan the specimens at a voltage of 55 kV and a current of 145 μA with a voxel size of 25 μm and a total scanning distance of 25.6 mm. On each two-dimensional tomogram, the cortical bone was masked out using a manually drawn contour. The resulting grey scale images were segmented using an adaptive threshold.

### 3.5. Biomechanical Testing

After sacrifice, both tibiae of each animal were prepared, and soft tissue was removed carefully. For biomechanical testing, the bones were fixed in a special device and preloaded with an axial force of 5 N. A constant linear propulsion (1 mm/min), generated by a material testing machine (Zwick 1455, Ulm, Germany), was applied to a lever arm attached to one of the pivoted axes for transforming the translation of the material-testing machine to a uniform torsional movement. The other side was connected with a load cell (*F*_max_ = 50 N, HBM, Darmstadt, Germany), which recorded the force. Maximum load and torsional stiffness was calculated. The values were expressed as the percentages of the contralateral intact tibia.

### 3.6. Histological Analysis

For histological evaluation, the soft tissue was removed from the entire tibia, taking care not to destroy the callus tissue. Bones were fixed for 48 h in 10% normal buffered formalin. Bone specimens taken at Day 10 were decalcified with ethylenediaminetetraacetate (EDTA), dehydrated, embedded in paraffin and longitudinal sections (4 μm; Leica SM 2500s microtome, Wetzlar, Germany) were made. Slices were stained with hematoxylin and eosin (HE) and Alcian blue. The samples of the later time points (Days 28–84) were embedded in polymethylmethacrylate (Technovit 9100 neu; Heraeus Kulzer, Wehrheim, Germany). Longitudinal sections (4 μm) were cut and stained with Safranin orange/van Kossa. For the evaluation, a region of interest (ROI) was defined, including the zone of reactive callus proximal and distal from the center of the osteotomy gap extended in length 1.5-fold of the individual cortical bone diameter. At Day 10, the reactive callus (HE), cartilaginous fraction (Alcian blue), as well as early woven bone (Movat pentachrome) was quantified. At the later time points, Days 28–84, the amount of mineralized and cartilaginous tissue (Safranin orange/van Kossa) was evaluated. Two image analyzing systems were used: (1) Image J for callus composition and cartilaginous fraction at Day 10; (2) KS 400; Zeiss, Göttingen for mineralized and non-mineralized tissue amount at Days 28, 42 and 84 Movat-Pentachrom staining was done for overview pictures.

A group size of *n* = 6 per time point for histology and biomechanical testing was planned. Due to death during anesthesia, implant dislocation or problems during the processing, 8 specimens were lost.

### 3.7. Statistical Analysis

The number of animals ranged between 4 and 9 animals per group, depending on the method and time point (Table 1). Animals of the control group were part of a previously conducted study [30], because the 3-R principle (replace, reduce and refine) is demanded for animal experiments. For the histomorphometry, new histological slices were stained and analyzed. For statistical comparison of the treatment groups, the Mann–Whitney U test for non-parametric data (PASW Statistics 18.0; SPSS, IBM, New York, NY, USA) was used. A *p*-value of less than 0.05 was taken as a significant difference. Values are given as medians and the 25%–75% percentile and whiskers represent minimum and maximum values.

## 4. Conclusions

Sustained BMP-2 application resulted in an improved bone healing with enhanced mineralization, remodeling and biomechanical stiffness compared to the control. Comparing the data from this study using a sustained, but incomplete release of BMP-2 (only approximately. 1 μg) to the previous study with the initial burst and complete release of BMP-2 (approximately. 40 μg), a comparable healing outcome could be detected. As a result, the sustained release of a much lower amount of BMP-2 had the same efficacy, as the high burst release. These results indicate the need to optimize the BMP-2 concentrations for sufficient stimulation of bone healing. Further work is necessary to modify release systems that meet the requirements for dosage and release kinetics.

## Figures and Tables

**Table 1.** Number of animals and investigated parameters of the osteotomized tibia at the different post-operative time points.

Group	Specimen per method and time point									

	μCT *				Histomorphometry			Biomechanics		
Day	10	28	42	84	28	42	84	28	42	84
Control	8	8	8	4	5	6	6	5	5	5
BMP-2	9	6	9	9	4	5	5	6	6	6

* The μCT imaging was made with animals used later for histological or biomechanical analysis.
